# NIPTRIC: an online tool for clinical interpretation of non-invasive prenatal testing (NIPT) results

**DOI:** 10.1038/srep38359

**Published:** 2016-12-05

**Authors:** Birgit Sikkema-Raddatz, Lennart F. Johansson, Eddy N. de Boer, Elles M. J. Boon, Ron F. Suijkerbuijk, Katelijne Bouman, Catia M. Bilardo, Morris A. Swertz, Martijn Dijkstra, Irene M. van Langen, Richard J. Sinke, Gerard J. te Meerman

**Affiliations:** 1 University of Groningen, University Medical Centre Groningen, Department of Genetics, Groningen, the Netherlands; 2 University of Groningen, University Medical Centre Groningen, Genomics Coordination Centre, Department of Genetics, Groningen, the Netherlands; 3 Department of Clinical Genetics, Laboratory for Diagnostic Genome Analysis, Leiden University Medical Centre, Leiden, the Netherlands; 4 University of Groningen, University Medical Centre Groningen, Department of Obstetrics and Gynaecology, Groningen, the Netherlands

## Abstract

To properly interpret the result of a pregnant woman’s non-invasive prenatal test (NIPT), her *a priori* risk must be taken into account in order to obtain her personalised *a posteriori* risk (PPR), which more accurately expresses her true likelihood of carrying a foetus with trisomy. Our aim was to develop a tool for laboratories and clinicians to calculate easily the PPR for genome-wide NIPT results, using diploid samples as a control group. The tool takes the *a priori* risk and Z-score into account. Foetal DNA percentage and coefficient of variation can be given default settings, but actual values should be used if known. We tested the tool on 209 samples from pregnant women undergoing NIPT. For Z-scores < 5, the PPR is considerably higher at a high *a priori* risk than at a low *a priori* risk, for NIPT results with the same Z-score, foetal DNA percentage and coefficient of variation. However, the PPR is effectively independent under all conditions for Z-scores above 6. A high PPR for low *a priori* risks can only be reached at Z-scores > 5. Our online tool can assist clinicians in understanding NIPT results and conveying their true clinical implication to pregnant women, because the PPR is crucial for individual counselling and decision-making.

Non-invasive prenatal testing (NIPT) for foetal aneuploidies, by analysing cell-free DNA in maternal blood, has been offered to pregnant women increasingly since 2011 [reviews refs 1, 2, 3]. Large clinical studies including about 150,000 pregnancies have reported a sensitivity and specificity for NIPT of more than 99% for foetal trisomy 13 or 21, and of 98% for trisomy 18 [refs 4 and 5, reviews refs 1 and 2]. This performance of NIPT in the general population of pregnant women^3^,^4^,^5^,^6^,^7^,^8^,^9^,^10^ appears to be similar for both low-risk and high-risk pregnancies^4^,^5^,^10^,^11^.

NIPT can identify pregnancies at risk for a trisomy and is therefore a screening tool, not a diagnostic test. For an individual woman, a positive NIPT result with a sensitivity and specificity of more than 99% does not mean that she actually has more than a 99% chance of carrying a foetus with a trisomy. Her true likelihood depends not only on her NIPT result, but also on the prevalence of the anomaly in the population she belongs to^12^, which is expressed as an *a priori* risk. Thus, her individual *a priori* risk for a specific foetal trisomy is based on her age, the gestational age at which NIPT is performed, and the results of other screening tests such as the first trimester combined test (FCT). The result of a NIPT for an individual woman in most of the genome-wide methods is calculated as a Z-score, where the individual sample is compared with a control group of normal (diploid) samples. However, presenting NIPT results to clinicians and pregnant women as “normal or abnormal” or as a Z-score makes it difficult for clinicians to interpret and use the result to correctly inform a pregnant woman of her true likelihood of carrying a foetus with a trisomy. In order to properly counsel women about a positive result from a cell-free foetal DNA screening, it can be useful to express the result as a personalised *a posteriori* risk (PPR), which takes the woman’s *a priori* risk into account.

Although not all cell-free foetal DNA screening providers calculate a Z-score or need *a priori* risks, it is important for women to know their true chance of carrying a Down syndrome foetus after a positive test. This chance might be far lower than that concluded from the Z-score percentile (e.g. 99%) that might otherwise be a reason for them to undergo a confirmatory amniocentesis. Knowing the true risk could help avoid a hasty and sometimes unnecessary termination of pregnancy^13^,^14^, or a pregnant woman being wrongly reassured by being given a negative NIPT result. Thus, in clinical counselling and decision-making following a (positive) NIPT result, the PPR is the most important factor for the parents.

NIPT is currently dominated by commercial testing providers. However, only a few of them provide the PPR with the NIPT result, nor is the calculation of the PPR published or straightforward for the clinician to understand^15^.

We have therefore developed a web-based tool to calculate the PPR according to the *a priori* risk (for trisomy 13, 18, 21) of the mother in combination with the outcome of her NIPT test, expressed as a Z-score. Our tool can easily be used by cell-free foetal DNA screening providers and healthcare professionals.

## Results

Our tool is freely available online (www.niptric.eu). To test the tool’s validity we calculated a PPR for a range of extreme values: for a specific *a priori* risk, given the observed Z-score but unknown percentage of foetal DNA and coefficient of variation (see also Supplementary Table 1). The PPR based on the observed Z-score and the known percentage of foetal DNA, at an assumed coefficient of variation of 0.5 and an *a priori* risk of 1:1000, 1:100, and 1:10 are given in Supplementary Tables 2, 3, and 4. However, in the online tool, the PPR can be calculated for every combination of the four parameters (*a priori* risk, observed Z-score, percentage of foetal DNA and coefficient of variation).

The use of the PPR calculator and its interpretation is illustrated here by three examples. We show how the PPR is calculated from the woman’s NIPT result to yield the likelihood of her carrying a foetus with Down syndrome: if she is at low risk (*a priori* risk of 1:1000), at high risk (*a priori* risk of 1:100), or at very high risk (*a priori* risk of 1:10). Here, the more general trends are shown for the impact of the four parameters.

**Figure 1:**
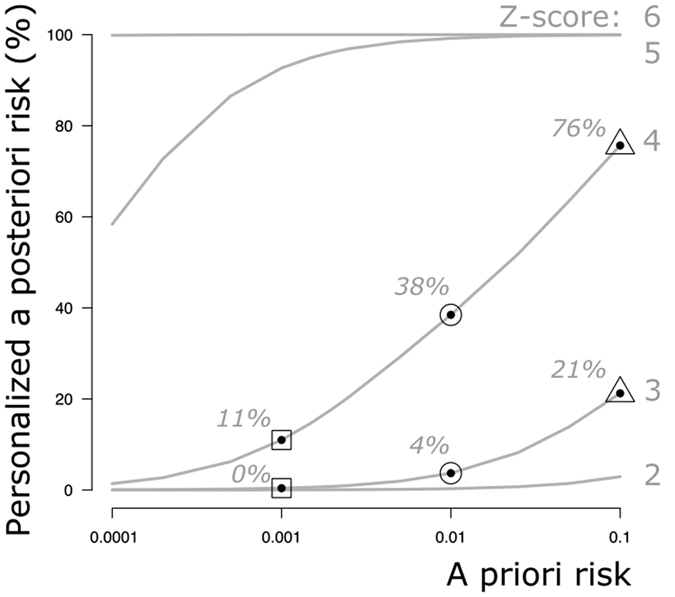
Estimation of the PPR for a specific *a priori* risk for a given observed Z-score and in the absence of information on the percentage of foetal DNA for a woman at low risk (1:1000) [square boxes], at high risk (1:100) [circles], and at very high risk (1:10) [triangles]. The PPR for the woman at low risk (1:1000 (0.001)) is <1% at a Z-score of 3, increasing to 11% for a Z-score of 4. This means that with a positive NIPT result, with a Z-score of 3 or 4, the actual chance of the woman carrying a foetus with Down syndrome is <1% or 11%, respectively. The woman at high risk (1:100 (0.01)) has a chance of 4% with a Z-score of 3, but a chance of 38% with a Z-score of 4. For the woman at very high risk (1:10 (0.1)), the PPR is 21% for a Z-score of 3 and 76% for a Z-score of 4.

Figure 1 shows the impact of variable *a priori* risk values and observed Z-scores on the PPR. The PPR increases when the Z-score increases and the woman has a higher *a priori* risk. Thus, the increase of PPR at a Z-score between 3 and 4 is more striking in high-risk pregnancies than in low-risk pregnancies. For a Z-score of 6 or higher, the PPR is approximately 100%, and is therefore effectively independent of the *a priori* risk (see Supplementary Table 1) for a given coefficient of variation and foetal DNA percentage value.

**Figure 2:**
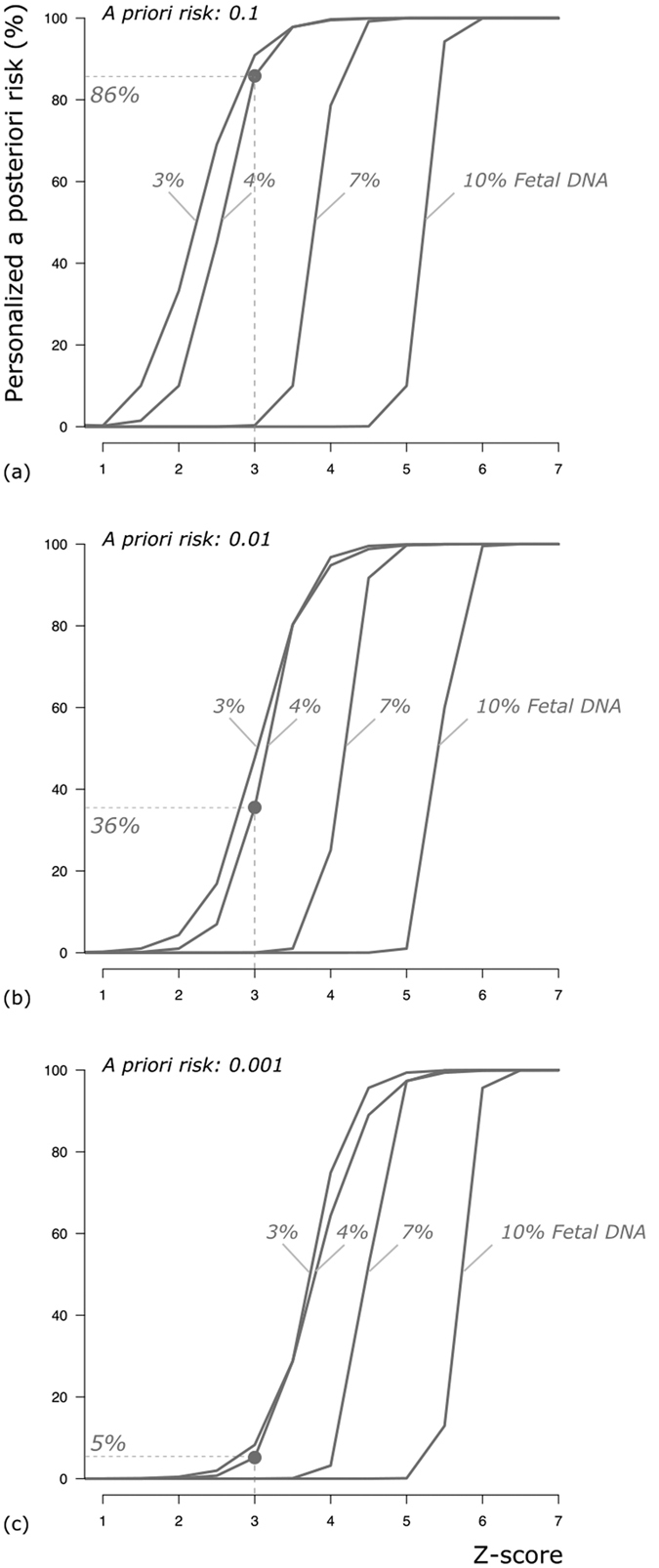
Estimation of the PPR given an observed Z-score and percentage of foetal DNA at a coefficient of variation of 0.5%. (**a**) *a priori* risk of 0.1 (1:10); (**b**) *a priori* risk of 0.01(1:100); and (**c**) *a priori* risk of 0.01(1:1000). x-axis: Z-score range 1–7. y-axis: Personalised *a posteriori* risk (%). After a positive NIPT result at a Z-score of 3 and at 4% foetal DNA, the low-risk woman has a 5% chance of carrying a foetus with Down syndrome and thus a 95% chance of the result being false-positive. In contrast, the higher-risk women have a 36% (1:100) and an 86% (1:10) chance of carrying a foetus with Down syndrome. Thus, the chance of a false-positive result at a risk of 1:100 and 1:10 is 64% and 14%, respectively.

Figure 2 illustrates the impact of different percentages of foetal DNA on the PPR for different *a priori* risks and according to the Z-scores. At a Z-score of 3, the percentage of false-positive results is much higher for a woman who is at low *a priori* risk (1:1000) than for one at higher risk (1:100 or 1:10). Figure 2 also shows that, with the given foetal DNA percentages, the chance of carrying a foetus with Down syndrome is >99% for both low-risk (1:1000) and high-risk (1:100 and 1:10) women if the Z-score is above 6 (see also Supplementary Tables 2, 3, and 4).

### Performance of the PPR calculator

The performance of our PPR calculator was tested in 209 samples. Of these 14 showed a Z-score > 3 (Table 1). In ten samples, the Z-score was > 6, resulting in a PPR of >99%. In four samples, a Z-score of between 4 and 6 was calculated, resulting in PPRs between 4–40%. In one of these samples, a mosaic trisomy 21 was confirmed in chorionic villi and amniotic fluid, while two samples had a normal diploid outcome in amniotic fluid. In the fourth sample, the parents refused invasive follow-up because of a PPR of 4% for trisomy 13. No abnormalities were seen on ultrasound at 16 weeks’ gestation and a healthy child was born.

## Discussion

We present an easy-to-use online tool to assist cell-free foetal DNA screening providers and healthcare professionals in calculating a woman’s PPR after a positive NIPT result. Our tool takes into account both test and patient characteristics. The online program can be used to estimate the PPR of any NIPT result according to a woman’s personal *a priori* risk and Z-score.

Some screening services offering NIPT use dedicated proprietary algorithms to calculate an individual risk figure^16^ or to discriminate between pregnancies with a low (<1%) or high risk (>1%) for a trisomy^17^; they take into account the combination of the Z- or likelihood score, *a priori* risk, and the percentage of foetal DNA in the NIPT test. However, most of these algorithms are not freely available and other services do not provide this essential information. Existing PPR calculators give only general information, such as sensitivity, specificity, positive predictive value, and *a priori* risk^18^,^19^. These numbers do not relate to the individual situation of a pregnant woman.

To satisfy the need for a woman’s personalised *a posteriori* risk figure, our PPR calculator can be applied to the results of genome-wide NIPT methods using diploid control samples. Different NIPT methods have been developed based on whole genome sequencing^20^,^21^ or on selected chromosome targeted-sequencing^22^,^23^. Most of these methods compare the individual sample with a population of normal (diploid) control samples, with the outcome usually presented as a Z-score. This can be based either on the difference of a number of single nucleotide polymorphisms^22^, or on a fraction of reads from whole genome sequencing^20^,^21^ or from targeted-sequencing^23^. Using this Z-score, the PPR can then be calculated in combination with the *a priori* risk in our calculator. If providers do not calculate an *a posteriori* risk they can easily add the PPR calculation using our tool as part of their service. Those healthcare professionals who only receive a Z-score as the outcome from a NIPT test can then use our tool together with the individual woman’s *a priori* risk to gain a more accurate *a posteriori* risk for counselling the individual woman or parents.

The outcome is still, of course, a risk estimation, not an exact number. Moreover, our PPR calculator may be applied to detect any aneuploidy provided that the *a priori* risk for the particular aneuploidy is known for the gestation period in which the NIPT is performed. However, a negative NIPT result may be falsely reassuring for women at high risk who also have nuchal translucency or ultrasound findings that cause concern if only chromosomes 13, 18 and 21 have been tested^24^.

For each woman, the PPR of a diagnostic or screening test depends on the prevalence of the disease in her population^25^. Accordingly, we have shown that, after a positive NIPT result, the PPR is also influenced by the individual’s risk profile. For Z-scores < 5, the PPR is considerably higher at a high *a priori* risk than at a low risk for a NIPT result with the same Z-score, coefficient of variation and foetal DNA percentage, while the PPR becomes effectively independent of these parameters for Z-scores > 6. A high PPR for a low *a priori* risk can only be reached at Z-scores > 5. In line with our calculations, Bianchi *et al*.^10^ demonstrated that even at a high sensitivity and specificity for NIPT, the positive predictive values for trisomy 21 and trisomy 18 in low- or average risk pregnancies were only 45% and 40%, respectively, which means that the PPR for an individual woman is, on average, also equal to this percentage. This was confirmed in a routine screening of a prenatal population (N = 15,841) with a positive predictive value of 80%^5^, while Wang *et al*.^26^ estimated values for the less common trisomy 18 and trisomy 13 at 64% and 44%, respectively, compared to 94% for trisomy 21. As Borrell and Stergiotou (2015) stated, some referring physicians may think that NIPT is a diagnostic test and they may not realise they also need take into account that the positive predictive value may vary strongly for individual women^27^. Some authors^12^,^17^ suggest that, at minimum, the *a priori* risk should be incorporated in assessing a NIPT result. Our calculations strongly support this suggestion.

Our PPR calculator can even be used when the coefficient of variation and the percentage of cell-free foetal DNA in the maternal plasma are unknown or not given. We included this option in our tool because some laboratories do not provide a foetal DNA percentage due to the difficulties in measuring samples in a pregnancy with a female foetus. At minimum, a Z-score and the *a priori* risk are needed as input for our tool, whereas default settings for the percentage of DNA and coefficient of variation can be used. However, several studies have shown that low percentages of foetal DNA in maternal plasma are related to test failures and false-negative results^28^,^29^. Thus, a lower limit of 4% foetal DNA was proposed as the cut-off for a reliable result^8^,^30^. Our online tool gives extra weight to the extreme values of the DNA foetal percentage in the population compared to a normal distribution to yield a higher PPR prediction in the presence of low percentages of foetal DNA. This is advantageous because the percentage of foetal DNA in maternal plasma might, in general, be lower for trisomy 13 and 18^8^,^31^,^32^,^33^. Nonetheless, in the ideal situation, the healthcare provider should also be given the coefficient of variation and percentage of foetal DNA, since these are important indicators for the sensitivity of NIPT. Use of the actual percentage of foetal DNA and coefficient of variation further improve the accuracy of the PPR calculation. Even when the percentage of foetal DNA is measured, a small range for the upper and lower limit is advisable because the measurement is not always precise. Without an estimation of the percentage of foetal DNA, we advise using 1% as the lower limit and 23% as the upper, which our tool has as default settings.

Computations using our PPR calculator with relatively low percentages of foetal DNA in maternal blood have shown two trends. First, a low percentage reduces the PPR far more in low-risk pregnancies than in high-risk ones, which could lead to more false-positive results. Second, in high-risk pregnancies, negative results are more likely to be false for Z-scores between 2 and 3 in combination with low percentages (<7%) (e.g. PPR of 45% at Z = 2.5, coefficient of variation 0.5, *a priori* risk 1:10, foetal percentage 4%). This is partly in line with Bianchi *et al*.^34^, who considered a Z-score between 2.5 and 4 as a borderline value.

Thus, false-negative results might be obtained if the actual percentage of foetal DNA is low and the coefficient of variation is higher than our default settings due to a lower sensitivity of the NIPT test. Measuring (and reporting) the percentage of foetal DNA^28^, and knowing the coefficient of variation, are therefore important prerequisites for the accurate interpretation of NIPT results^21^,^35^ now that easy-to-use methods are available^36^.

False-positive results can also be obtained, because the NIPT result might only reflect the genetic status of the placenta and not that of the foetus due to confined placenta mosaicism^37^,^38^,^39^. This is relevant in trisomy 21, which results in a larger standard variation for trisomic samples. To avoid this problem, we recommend using a larger range for the lower and upper values of the foetal DNA percentage. Our tool calculates the risk of a non-mosaic trisomy. Thus, a Z-score that is lower than expected for a specific foetal percentage, but higher than expected for an euploid sample, might indicate the presence of mosaicism. In general, a positive NIPT result, even with a posterior risk of >99%, should always be confirmed with amniocentesis.

In conclusion, our PPR calculator can be easily used by cell-free foetal DNA screening providers and healthcare professionals to interpret NIPT results obtained by genome-wide methods. We urge them to use our tool in making further clinical decisions. The calculation of the PPR stresses the importance of confirming a positive NIPT result by invasive prenatal diagnosis, because not every pregnant woman with a positive result has the same likelihood of carrying a foetus with an aneuploidy. Our online software tool, figures and tables will help professionals and patients to better understand NIPT results and their implications in clinical practice.

## Material and Methods

### The PPR calculator

The PPR for a foetal trisomy (13, 18, or 21) for an individual pregnancy is estimated using four input parameters. By combining the *a priori* risk (calculated based on the mother’s age and gestation, or based on other screening tests) with the individual NIPT result (computed as a Z-score), the percentage of foetal DNA and the coefficient of variation of the control group, our tool can be used to calculate a meaningful personalised posterior risk (PPR) to aid interpretation of an individual NIPT result.

#### A priori risk

There are generally accepted risk tables for the population-based prevalence of trisomy 21^40^, trisomy 18, and trisomy 13^41^. These tables are used in the PPR calculator, if necessary, using bivariate linear interpolation, to calculate the *a priori* risk from the maternal age in combination with the gestational age at which the NIPT was performed. If the risk has been determined based on a first trimester combined test (FCT) or a previous child with a trisomy, this risk should be used because it reflects the individual *a priori* risk more precisely.

#### Z-score

The result of a NIPT for an individual woman is expressed as a Z-score, where the individual sample is compared with a control group of normal (diploid) samples. In the case of an aneuploidy of a chromosome, a relative excess or deficit for that chromosome is present compared to the normal diploid situation. A Z-score represents the number of standard deviations that the sample fraction of that chromosome deviates from the mean measured in normal (diploid) pregnancies assessed by a Gaussian distribution. The distinction is based on the statistical assumption that 99.7% of the plasma samples derived from pregnant women with a diploid foetus give a Z-score between −3 and +3. Thus, the more the Z-score deviates from zero, the more the individual sample deviates from the control group and thus points towards an aneuploidy.

The higher value of the Z-score for aneuploid samples, and thus the reliability of NIPT, however, depends on the assay precision which, in turn, depends on a number of factors such as the number of reads, the reference samples chosen, the method of sample preparation, and sequencing method. All these factors are encompassed in the coefficient of variation of control samples and, together with the percentage of foetal DNA in the maternal plasma^42^, they influence the Z-score.

#### Percentage of foetal DNA

The percentage of foetal DNA is essential to understanding the strengths and limitations of NIPT^8^,^43^ and it is a key factor in the NIPT procedure. For low percentages of foetal DNA, the distribution curves of the diploid and aneuploid fractions will overlap, as demonstrated by Benn and Cuckle^43^. In principle, a low percentage of foetal DNA will result in a low Z-score for a trisomic sample. The percentage of foetal DNA at a gestational age between 12 and 23 weeks (a median of 16 weeks) shows roughly a normal distribution between 1–23%, with outliers between 23–30%^44^,^45^. The mean measured foetal fraction for all samples is 12%. The rationale behind our default setting, which can be used when the percentage of foetal DNA is unknown, is to mimic a normal distribution with extra weight for the extreme values. We therefore chose a combination of two uniform distributions, one between 1–23% and another between 6–18% foetal DNA, with respective weights of 0.4 and 0.6. A few samples will have such extreme values (<6% or >18%). A low percentage of foetal DNA is the most critical parameter for calculating a low Z-score in aneuploidy samples and, if the percentage has been measured, it is known to only approximate score accuracy. A more precise prediction can be obtained by filling in the lower and upper limits of the measured foetal percentage in our tool.

Due to the extra weight given to low foetal percentages, the PPR will be higher than that calculated for actual percentages of foetal DNA lying between 1–6% compared to a normal distribution.

#### Coefficient of variation

The random variability of the test is measured as the coefficient of variation of the control group. The coefficient will increase as the assay precision decreases, depending on the quality of the laboratory procedure, i.e. sample preparation or number of reads. Increasing the number of reads can improve the assay precision and thus reduce the coefficient of variation of the control group. Different algorithms have been developed to increase precision, by reducing the variation in the control group, e.g. GC correction^46^, or by using an adapted Z-score calculation, such as the normalised chromosome value^35^,^47^. The coefficient of variation is used in combination with the percentage of foetal DNA to compute the expected distance between the two Gaussian distributions for diploid pregnancies and trisomic pregnancies. The calculation is made as follows:


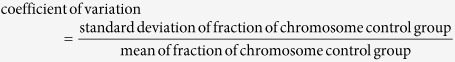


As the coefficient of variation increases, the distance between the diploid and aneuploid distribution will decrease, resulting in a decrease of sensitivity for detecting a trisomy. For example, a coefficient of variation of 0.5% for chromosome 21 would result in 99.87% sensitivity at a foetal DNA percentage of 6%, while the sensitivity would drop to 84.13% at a foetal DNA percentage of 4%. A 99.87% sensitivity at 4% foetal DNA can only be obtained with a coefficient of variation of 0.33%. Thus, a higher coefficient of variation will decrease the sensitivity, especially at low percentages of foetal DNA. A coefficient of variation of 0.5% (chromosome 21) is used as the default setting in our program because this is close to empirically measured values. For chromosomes 13 and 18, we recommend 0.4% as a default setting. The number of reads is higher for these chromosomes, leading to the expectation of a lower coefficient of variation than for chromosome 21. However, if the coefficient of variation is measured and lower than our default setting, this value should be used for a more accurate PPR calculation.

The PPR calculation is made as follows. First, the expected Z-score, if a trisomy is present, is calculated using the coefficient of variation of the control group and the percentage of foetal DNA:


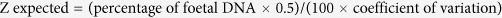


The actual Z-score in the case of a trisomy is a random variable with the “Z expected” value and standard deviation both equal to 1.0. Because the percentage of foetal DNA cannot be exactly measured, the empirical distribution of Z-scores will be a weighted sum of distributions over all possible values for the foetal DNA percentage. Technically, this percentage is a nuisance parameter that is integrated out to compute the probability that the observed Z-score originates from a trisomic pregnancy. In our computational model, we allow the range for the foetal DNA percentage to be known and input exactly. The actual integration of the nuisance parameter of foetal percentage is done by converting the foetal DNA percentage to a lower and upper value for the expected Z- score.

The post-test probability or personalised *a posteriori* risk (PPR) is calculated as:


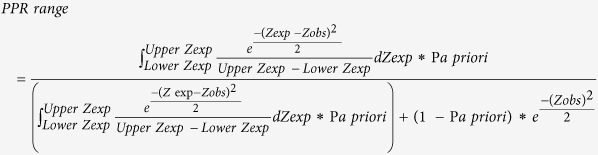



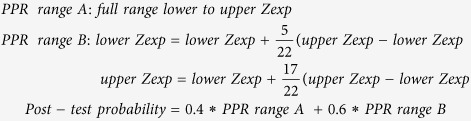


### Examples of the use of the PPR calculator

To demonstrate the use of the calculator and the effects of varying *a priori* risk values and observed Z-scores on the PPR, we have generated tables and concomitant figures. In order to clarify the calculations, we fixed the coefficient of variation at 0.5 and used a range of 1–23% of cell-free foetal DNA. The PPR was calculated as a percentage for *a priori* risks of 0.0001, 0.0002, 0.0005, 0.0010, 0.0015, 0.0020, 0.0025, 0.0050, 0.0100, 0.0250, 0.0500 and 0.1000, for observed Z-scores of 2, 2.5, 3, 3.5, 4, 4.5, 5, 5.5 and 6.

To demonstrate the additional effect on the PPR of variable foetal DNA percentages in maternal blood, the PPR was calculated for an *a priori* risk of 0.001, 0.01 and 0.1, for Z-scores varying from 0 to 7 and foetal DNA varying from 3% to 10%.

### Performance of the PPR calculator

To test the performance of our PPR calculator, we analysed 209 maternal blood samples obtained from pregnant women with an elevated risk for trisomy 13, 18 or 21 due to an FTC > 1:200 between 10 and 16 weeks of gestation. This was part of the trial by Dutch laboratories for evaluation of non-invasive prenatal testing (TRIDENT) program, and supported by the Dutch Ministry of Health, Welfare and Sport (11016-118701-PG). The trial was conducted according prescribed laboratory protocols. Our study was approved by the Ethics Committee of the University Medical Centre Groningen. All participants signed an informed consent form.

Data were obtained from massively parallel, shotgun sequencing of cell-free DNA from maternal plasma with a Solid Wildfire sequencing system (Life Technologies Ltd., Paisley, UK). The sequencing data were used to calculate a Z-score. For the calculation of the PPR, we used as input the *a priori* risk as determined at FTC, the Z-score, the actual coefficient of variation, and the default setting for the percentage of foetal DNA. The outcome of the NIPT was either confirmed in amniotic fluid by karyotyping or by follow-up after birth.

## Additional Information

**How to cite this article**: Sikkema-Raddatz, B. *et al*. NIPTRIC: an online tool for clinical interpretation of non-invasive prenatal testing (NIPT) results. *Sci. Rep.* **6**, 38359; doi: 10.1038/srep38359 (2016).

**Publisher's note:** Springer Nature remains neutral with regard to jurisdictional claims in published maps and institutional affiliations.

## Supplementary Material

Supplementary Datasets

## Figures and Tables

**Table 1:** Summary of all samples with a Z-score > 3 for chromosomes 13, 18 or 21 in 209 samples on which NIPT was performed.

Sample	First trimester combined test risk for Trisomy 21	Coefficient of variation #21	Z-score #21	Posterior Risk (%)	Confirmation by karyotyping in amniotic fluid
1	1/4	0.40	13.7	99.9	47#, +21
2	1/2	0.29	27.2	99.9	47#, +21
3	1/79	0.31	12.4	99.9	47#, +21
4	1/118	0.40	11.6	99.9	47#, +21
5	1/141	0.33	14.4	99.9	47#, +21
6	1/119	0.47	11.9	99.9	47#, +21
7	1/13	0.32	19.7	99.9	47#, +21
8	1/20	0.36	16.9	99.9	47#, +21
9	1/115	0.29	26.2	99.9	47#, +21
10	1/25	0.33	28.8	99.0	47#, +21
11	1/43	0.33	4.9	40.0	Mos 46#/47#, +21 also seen in chorionic villi
12	1/147	0.34	4.4	36.0	46#, no T21
13	1/80	0.32	4.2	33.0	46#, no T21
*14	#13 1:5000	#13 0.18	#13 4.4	4.0	No confirmation done, healthy baby born

^*^A Z-score of 4.4 for chromosome 13 was detected, while the *a priori* risk for trisomy 21 was 1/121; no elevated risk was found for trisomy 13 after the first trimester combined test.
