# Supplementary material for: NIPTRIC: an online tool for clinical interpretation of non-invasive prenatal testing (NIPT) results
Source: Sci Rep. 2016 Dec 5;6:38359. doi: 10.1038/srep38359 (PMC5137038; doi:10.1038/srep38359)
Supplement: Supplementary Datasets [file srep38359-s1.pdf]

**NIPTRIC: an online tool for clinical interpretation of non-invasive prenatal testing (NIPT) results**

Birgit SIKKEMA-RADDATZ[1,*], Lennart F. JOHANSSON[1,2] , Eddy N. de BOER[1], Elles M.J. BOON[3], Ron F. SUIJKERBUIJK[1], Katelijne BOUMAN[1], Catia M. BILARDO[4], Morris A. SWERTZ[2], Martijn DIJKSTRA[2], Irene M. van LANGEN[1], Richard J. SINKE[1], Gerard J. te MEERMAN[1]

## Supplementary tables

**Supplementary Table 1**. Estimation of the personalized *a posteriori* risk (PPR) for a specific *a priori risk,* given an observed Z-score and unknown percentage of fetal DNA

| ***A priori* risk** | Observed Z-score (%) | | | | | | | | |
|---|---|---|---|---|---|---|---|---|---|
| | 2 | 2.5 | 3 | 3.5 | 4 | 4.5 | 5 | 5.5 | 6.0 |
| 0.0001 | 0 | 0.01 | 0.04 | 0.21 | 1.40 | 10.88 | 58.39 | 96.66 | 99.86 |
| 0.0002 | 0.01 | 0.02 | 0.08 | 0.42 | 2.71 | 18.41 | 72.79 | 99.30 | 99.93 |
| 0.0005 | 0.01 | 0.05 | 0.20 | 1.03 | 6.23 | 32.39 | 86.53 | 99.31 | 99.97 |
| 0.001 | 0.03 | 0.10 | 0.40 | 2.01 | 10.98 | 45.11 | 92.68 | 99.66 | 99.99 |
| 0.0015 | 0.04 | 0.15 | 0.60 | 2.94 | 14.75 | 53.01 | 94.97 | 99.77 | 99.99 |
| 0.002 | 0.06 | 0.19 | 0.79 | 3.83 | 17.84 | 58.69 | 96.17 | 99.83 | 99.99 |
| 0.0025 | 0.07 | 0.24 | 0.98 | 4.68 | 20.44 | 63.04 | 96.91 | 99.86 | 99.99 |
| 0.005 | 0.14 | 0.48 | 1.93 | 8.44 | 29.20 | 75.57 | 98.43 | 99.93 | 100.00 |
| 0.01 | 0.28 | 0.96 | 3.69 | 14.09 | 38.47 | 85.38 | 99.21 | 99.97 | 100.00 |
| 0.025 | 0.71 | 2.34 | 08.22 | 23.71 | 51.86 | 93.42 | 99.70 | 99.99 | 100.00 |
| 0.05 | 1.44 | 4.52 | 13.89 | 31.08 | 63.51 | 96.63 | 99.85 | 99.99 | 100.00 |
| 0.1 | 2.92 | 8.48 | 21.24 | 37.78 | 75.66 | 98.36 | 99.93 | 100.00 | 100.00 |

**Supplementary table 2**. Estimation of the personalized *a posteriori* risk (PPR) based on an observed Z-score and the percentage of fetal DNA, at a coefficient of variation of 0.5 and an *a priori* risk of 1:1000

| Observed Z-score | Percentage of fetal DNA (%) | | | | | | | |
|---|---|---|---|---|---|---|---|---|
| | 3 | 4 | 5 | 6 | 7 | 8 | 9 | 10 |
| 0 | 0 | 0 | 0 | 0 | 0 | 0 | 0 | 0 |
| 0.5 | 0.01 | 0 | 0 | 0 | 0 | 0 | 0 | 0 |
| 1.0 | 0.02 | 0 | 0 | 0 | 0 | 0 | 0 | 0 |
| 1.5 | 0.10 | 0.01 | 0 | 0 | 0 | 0 | 0 | 0 |
| 2.0 | 0.45 | 0.10 | 0.01 | 0. | 0 | 0 | 0 | 0 |
| 2.5 | 1.97 | 0.73 | 0.01 | 0.01 | 0 | 0 | 0 | 0 |
| 3.0 | 8.27 | 5.18 | 1.21 | 0.10 | 0 | 0. | 0 | 0. |
| 3.5 | 28.77 | 28.77 | 12.94 | 1.97 | 0.10 | 0 | 0 | 0 |
| 4.0 | 64.41 | 74.90 | 64.41 | 28.77 | 3.21 | 0.10 | 0 | 0 |
| 4.5 | 89.02 | 95.66 | 95.66 | 89.02 | 52.33 | 5.18 | 0.10 | 0 |
| 5.0 | 97.32 | 99.39 | 99.63 | 99.39 | 97.32 | 74.90 | 8.27 | 0.10 |
| 5.5 | 99.39 | 99.92 | 99.97 | 99.97 | 099.92 | 99.39 | 89.03 | 12.94 |
| 6.0 | 99.86 | 99.99 | 100.00 | 100.00 | 100.00 | 99.99 | 99.86 | 95.66 |
| 6.5 | 99.97 | 100.00 | 100.00 | 100.00 | 100.00 | 100.00 | 100.00 | 99.97 |
| 7.0 | 99.99 | 100.00 | 100.00 | 100.00 | 100.00 | 100.00 | 100.00 | 100.00 |

**Supplementary table 3**. Estimation of the personalized *a posteriori* risk (PPR) based on an observed Z-score and the percentage of fetal DNA, at a coefficient of variation of 0.5 and an *a priori* risk of 1:100

| Observed Z-score | Percentage of fetal DNA (%) | | | | | | | |
|---|---|---|---|---|---|---|---|---|
| | 3 | 4 | 5 | 6 | 7 | 8 | 9 | 10 |
| 0 | 0.01 | 0 | 0 | 0 | 0 | 0 | 0 | 0 |
| 0.5 | 0.05 | 0 | 0 | 0 | 0 | 0 | 0 | 0. |
| 1.0 | 0.23 | 0.02 | 0 | 0 | 0 | 0 | 0 | 0. |
| 1.5 | 1.00 | 0.14 | 0.01 | 0 | 0 | 0 | 0 | 0 |
| 2.0 | 4.33 | 1.00 | 0.08 | 0 | 0 | 0 | 0 | 0 |
| 2.5 | 16.87 | 6.95 | 1.00 | 0.05 | 0 | 0 | 0 | 0 |
| 3.0 | 47.62 | 35.55 | 10.96 | 1.00 | 0.03 | 0 | 0 | 0 |
| 3.5 | 80.30 | 80.30 | 59.99 | 16.87 | 1.00 | 0.02 | 0 | 0 |
| 4.0 | 94.81 | 96.79 | 94.81 | 80.30 | 25.07 | 1.00 | 0.01 | 0 |
| 4.5 | 98.79 | 99.55 | 99.55 | 98.79 | 91.72 | 35.55 | 1.00 | 0.01 |
| 5.0 | 99.73 | 99.94 | 99.96 | 99.94 | 99.73 | 96.79 | 47.62 | 1.00 |
| 5.5 | 99.94 | 99.99 | 100.00 | 100.00 | 99.99 | 99.94 | 98.79 | 59.99 |
| 6.0 | 99.99 | 100.00 | 100.00 | 100.00 | 100.00 | 100.00 | 99.99 | 99.55 |
| 6.5 | 100.00 | 100.00 | 100.00 | 100.00 | 100.00 | 100.00 | 100.00 | 100.00 |
| 7.0 | 100.00 | 100.00 | 100.00 | 100.00 | 100.00 | 100.00 | 100.00 | 100.00 |

**Supplementary table 4**. Estimation of the personalized *a posteriori* risk (PPR) based on an observed Z-score and the percentage of fetal DNA, at a coefficient of variation of 0.5 and an *a priori* risk of 1:10

| Observed Z-score | Percentage of fetal DNA (%) | | | | | | | |
|---|---|---|---|---|---|---|---|---|
| | 3 | 4 | 5 | 6 | 7 | 8 | 9 | 10 |
| 0 | 0.12 | 0 | 0 | 0 | 0 | 0 | 0 | 0 |
| 0.5 | 0.55 | 0.03 | 0 | 0 | 0 | 0 | 0 | 0 |
| 1.0 | 0.24 | 0.20 | 0.01 | 0 | 0 | 0 | 0 | 0 |
| 1.5 | 10.00 | 1.48 | 0.08 | 0 | 0 | 0 | 0 | 0 |
| 2.0 | 33.24 | 10.00 | 0.90 | 0.03 | 0 | 0 | 0 | 0 |
| 2.5 | 69.06 | 45.09 | 10.00 | 0.55 | 0.01 | 0 | 0 | 0 |
| 3.0 | 90.91 | 85.85 | 57.51 | 10.00 | 0.33 | 0 | 0 | 0 |
| 3.5 | 97.82 | 97.82 | 94.28 | 69.06 | 10.00 | 0.20 | 0 | 0 |
| 4.0 | 99.51 | 99.70 | 99.51 | 97.82 | 78.63 | 10.00 | 0.12 | 0 |
| 4.5 | 99.89 | 99.96 | 99.96 | 99.89 | 99.19 | 85.85 | 10.00 | 0.08 |
| 5.0 | 99.98 | 99.99 | 100.00 | 99.99 | 99.98 | 99.70 | 90.91 | 10.00 |
| 5.5 | 99.99 | 100.00 | 100.00 | 100.00 | 100.00 | 99.99 | 99.89 | 94.28 |
| 6.0 | 100.00 | 100.00 | 100.00 | 100.00 | 100.00 | 100.00 | 100.00 | 99.96 |
| 6.5 | 100.00 | 100.00 | 100.00 | 100.00 | 100.00 | 100.00 | 100.00 | 100.00 |
| 7.0 | 100.00 | 100.00 | 100.00 | 100.00 | 100.00 | 100.00 | 100.00 | 100.00 |
